# Prospective Analysis of Antibody Diagnostic Tests and TTS1 Real-Time PCR for Diagnosis of Melioidosis in Areas Where It Is Endemic

**DOI:** 10.1128/jcm.01605-22

**Published:** 2023-03-06

**Authors:** Chawitar Noparatvarakorn, Sineenart Sengyee, Atchara Yarasai, Rungnapa Phunpang, Adul Dulsuk, Orawan Ottiwet, Rachan Janon, Chumpol Morakot, Mary N. Burtnick, Paul J. Brett, T. Eoin West, Narisara Chantratita

**Affiliations:** a Department of Microbiology and Immunology, Faculty of Tropical Medicine, Mahidol University, Bangkok, Thailand; b Department of Medical Technology and Clinical Pathology, Mukdahan Hospital, Mukdahan, Thailand; c Department of Medicine, Mukdahan Hospital, Mukdahan, Thailand; d Department of Microbiology and Immunology, University of Nevada, Reno School of Medicine, Reno, Nevada, USA; e Division of Pulmonary, Critical Care & Sleep Medicine, Harborview Medical Center, University of Washington, Seattle, Washington, USA; f Mahidol-Oxford Tropical Medicine Research Unit, Faculty of Tropical Medicine, Mahidol University, Bangkok, Thailand; Mayo Clinic

**Keywords:** *Burkholderia pseudomallei*, melioidosis, rapid immunochromatography test, serodiagnosis, PCR, TTS1, Hcp1, ELISA for melioidosis

## Abstract

Melioidosis is a tropical infectious disease caused by Burkholderia pseudomallei. Melioidosis is associated with diverse clinical manifestations and high mortality. Early diagnosis is needed for appropriate treatment, but it takes several days to obtain bacterial culture results. We previously developed a rapid immunochromatography test (ICT) based on hemolysin coregulated protein 1 (Hcp1) and two enzyme-linked immunosorbent assays (ELISAs) based on Hcp1 (Hcp1-ELISA) and O-polysaccharide (OPS-ELISA) for serodiagnosis of melioidosis. This study prospectively validated the diagnostic accuracy of the Hcp1-ICT in suspected melioidosis cases and determined its potential use for identifying occult melioidosis cases. Patients were enrolled and grouped by culture results, including 55 melioidosis cases, 49 other infection patients, and 69 patients with no pathogen detected. The results of the Hcp1-ICT were compared with culture, a real-time PCR test based on type 3 secretion system 1 genes (TTS1-PCR), and ELISAs. Patients in the no-pathogen-detected group were followed for subsequent culture results. Using bacterial culture as a gold standard, the sensitivity and specificity of Hcp1-ICT were 74.5% and 89.8%, respectively. The sensitivity and specificity of TTS1-PCR were 78.2% and 100%, respectively. The diagnostic accuracy was markedly improved if the Hcp1-ICT results were combined with TTS1-PCR results (sensitivity and specificity were 98.2% and 89.8%, respectively). Among patients with initially negative cultures, Hcp1-ICT was positive in 16/73 (21.9%). Five of the 16 patients (31.3%) were subsequently confirmed to have melioidosis by repeat culture. The combined Hcp1-ICT and TTS1-PCR test results are useful for diagnosis, and Hcp1-ICT may help identify occult cases of melioidosis.

## INTRODUCTION

Melioidosis is an often severe subtropical and tropical infectious disease that is endemic in northern Australia and Southeast Asia. The causative agent of melioidosis is the Gram-negative tier 1 select agent Burkholderia pseudomallei, which is naturally found in moist soils and surface waters in these areas (1). The routes of infection in humans include inhalation of aerosols of contaminated soil and dust, percutaneous inoculation, and ingestion of contaminated food and water (1). Worldwide, melioidosis cases have been estimated at 165,000 per year, with estimated deaths of 89,000 (54%) (2). In northeast Thailand, there are approximately 2,000 cases per year with a mortality rate exceeding 40%. Melioidosis has emerged in previously unaffected regions, such as North America and northeastern Brazil. This could be partially attributable to increased knowledge of the disease and improved diagnostic tools (1).

Melioidosis exhibits a broad spectrum of clinical symptoms that vary from localized cutaneous manifestations to severe sepsis and death (1). Bacteremia occurs in 40 to 60% of melioidosis patients, with approximately 20% of patients developing septic shock, the most severe form of melioidosis (1). Early diagnosis is required, since the optimal treatment requires intravenous administration of ceftazidime or a carbapenem, drugs that may not be widely available in regions where it is endemic. The median time for treatment response can be slow, up to 9 days (1).

For decades, diagnosis of melioidosis depended on isolation of bacterial culture, which requires microbiology facilities and can take several days. While bacterial culture is the diagnostic gold standard test for melioidosis, it is recognized to be imperfect, with Bayesian latent class modeling estimating the sensitivity to be 60% and the negative predictive value to be 61.9% (3). Even after isolation of bacteria, it can take several additional days to positively identify the organism, delaying diagnosis and appropriate treatment (4). Because of this, more rapid and non-culture-based diagnostics are desirable.

A point-of-care (POC) lateral flow assay for antigen detection was developed using a monoclonal antibody to B. pseudomallei capsular polysaccharide (CPS). However, the sensitivity was only 40%, based on evaluation with stored whole blood samples (5). PCR with specific primers can provide more rapid results. However, these assays need to overcome several challenges in clinical specimens, including low numbers of bacteria in whole blood (4) and PCR inhibitors present in the samples, such as immunoglobulin G (IgG), heme, and human leukocyte DNA (6, 7). Among several PCR targets, open reading frame 2 of the type three secretion system 1 (TTS1-*orf2*) gene cluster in B. pseudomallei is well-validated using real-time PCR for species-specific assays with clinical, animal, and environmental DNA samples from the Americas, Asia, Europe, Africa, and Oceania (8–11). However, performing the assay on buffy coat samples from Thai patients resulted in high specificity (100%) but low sensitivity (0%) (10). The sensitivity of the assay in buffy coat samples from Australia improved from 36% to 56% by increasing the volume of DNA samples (11).

Several serological tests have been developed and evaluated for the diagnosis of melioidosis. The indirect hemagglutination assay (IHA), which detects rising antibody titers against B. pseudomallei, is widely used but has low sensitivity and specificity in areas of endemicity (12, 13). Rapid enzyme-linked immunosorbent assays (ELISAs) targeting O-polysaccharide (OPS) and hemolysin coregulated protein 1 (Hcp1) associated with the B. pseudomallei type VI secretion system were developed and evaluated for serodiagnosis of melioidosis in an area of endemicity of Thailand (14, 15). The diagnostic accuracy of both ELISAs was significantly higher than that with the IHA (15). When anti-Hcp1 IgM and IgG antibodies were compared, an area under the receiver operating characteristic curve (AUROCC) was significantly greater for IgG (0.90) than for IgM (0.60) (16). Based on these promising results, a rapid immunochromatography test for POC detection of IgG antibodies to Hcp1 was recently developed (Hcp1-ICT). The diagnostic characteristics of the Hcp1-ICT were initially evaluated using Thai and U.S. serum samples and compared to bacterial culture results as the gold standard. Results demonstrated 88.3% sensitivity in Thai melioidosis patients, 86.1% specificity in Thai healthy donors, and 100% specificity in U.S. donors (17). More recently the Hcp1-ICT was evaluated in patients admitted to hospitals in northeast Thailand with a febrile illness but negative blood cultures. The specificity was determined to be 60.2%, with 39.8% of patients presenting Hcp1-ICT-positive results (unpublished data). This finding suggests that some Hcp1-ICT-positive but culture-negative patients may have undiagnosed melioidosis, previous exposure to B. pseudomallei, or cross-reactivity.

In the present study, we hypothesized that Hcp1 antibody detection might be a clinically useful tool for diagnosing melioidosis. To test this, we enrolled suspected melioidosis patients from northeast Thailand to validate the diagnostic accuracy of the Hcp1-ICT as a POC test for melioidosis using whole blood samples and compared the results with the results of bacterial culture, TTS1 real-time PCR, Hcp1-ELISA, and OPS-ELISA. We also evaluated the potential of Hcp1-ICT to identify occult cases of melioidosis. Occult cases were deemed likely to be melioidosis using combined results of repeated culture, real-time PCR, diagnostic imaging, and clinical investigations.

## MATERIALS AND METHODS

### Ethical approval.

The study protocol and related documents were approved by the Human Research Ethics Committees of the Faculty of Tropical Medicine, Mahidol University (approval number MUTM 2019-064-01) and of Mukdahan Hospital (MEC 03/62). The study was conducted in accordance with the Declaration of Helsinki and the principles of good clinical practice. Written informed consent was obtained from all patients.

### Study design and participants.

A prospective observational study was conducted at Mukdahan Hospital in Mukdahan Province, northeast Thailand. Adult patients with suspected melioidosis were recruited within 24 h of hospital admission (day 1) between October 2019 and November 2020. Suspected melioidosis cases were identified and selected by clinicians, including patients admitted to the hospital who met the criteria documented in the medical record or with clinical suspicion of melioidosis based on the following criteria: (i) sepsis, defined as an infection with organ dysfunction in accordance with the Third International Consensus (Sepsis-3) guidelines for sepsis (18, 19); (ii) patients without sepsis but with one of the following, fever (>38°C) or low temperature (<36°C) with any of the following diseases, diabetes mellitus (underlying disease or first diagnosis based on American Diabetes Association criteria [20, 21]), chronic kidney disease (22), or thalassemia. Exclusion criteria were admission to other hospitals with a total of admission time of >72 h, pregnancy, receiving palliative care, or incarceration. Vital status data were collected by follow-up phone calls conducted 28 days after admission. All blood and other clinical samples were collected on day 1 (the first day of hospital admission) for bacterial culture, Hcp1-ICT, TTS1 real-time PCR, Hcp1-ELISA, and OPS-ELISA.

**FIG 1:**
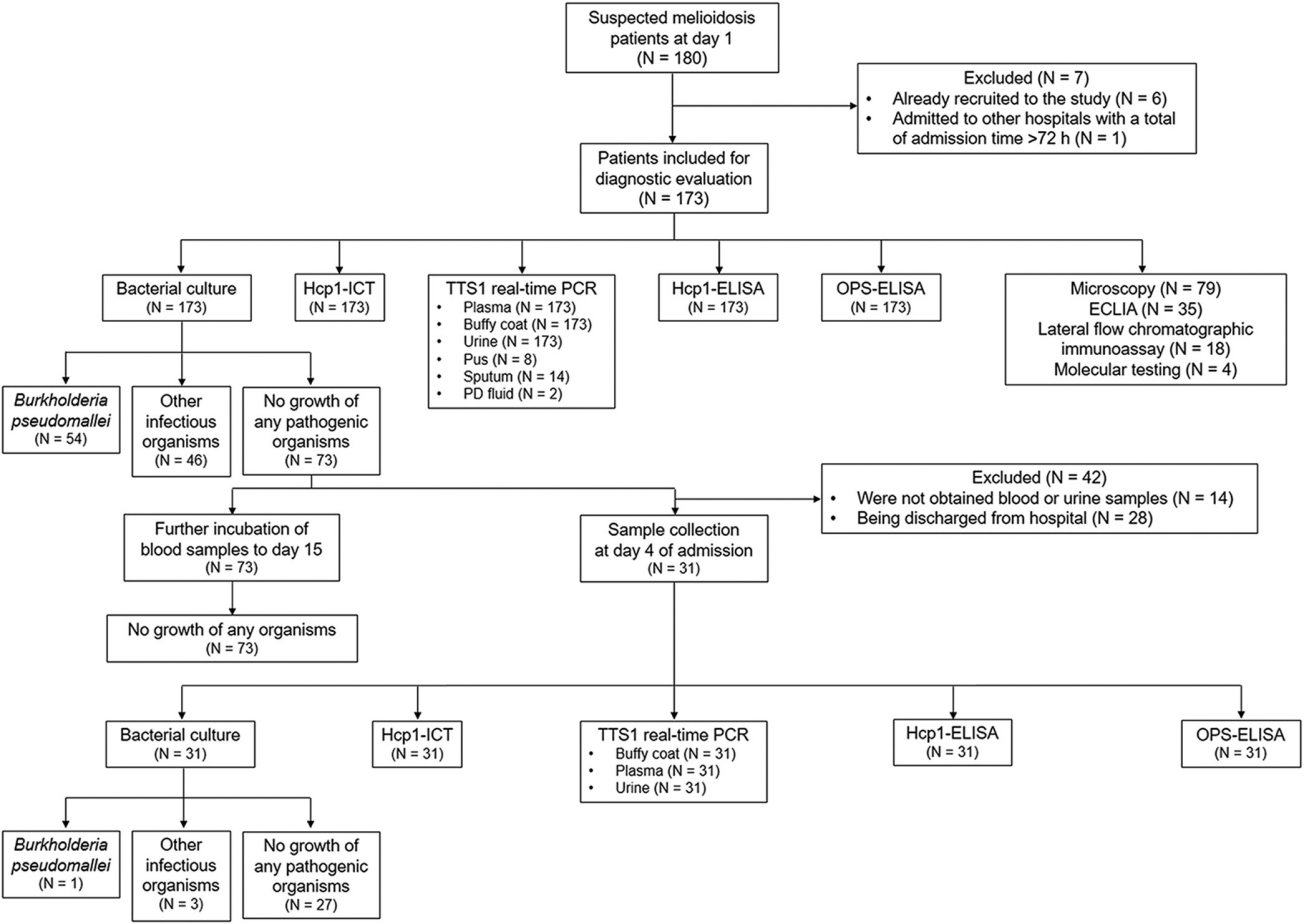
Diagnostic test evaluation flow chart. Clinical samples were collected from suspected melioidosis cases for assays on the day of admission (day 1). Patients whose day 1 cultures were negative underwent repeat blood and urine sampling at day 4 to investigate occult cases of melioidosis.

Pus, sputum, urine, and body fluid samples obtained for culture were incubated for 2 days. Blood cultures were performed using BacT/Alert 3D (bioMérieux, Marcy l'Étoile, France) and routinely incubated for 5 days. Patients with negative cultures and no other infection identified by other standard testing were further investigated as potentially occult cases of melioidosis. Clinical samples were collected from these patients for a second time on day 4 to repeat bacterial culture, Hcp1-ICT, TTS1 real-time PCR, Hcp1-ELISA, and OPS-ELISA (Fig. 1). Blood culture bottles with no growth results after 5 days were further incubated for a total of 15 days, and any bacteria detected were identified.

Confirmed melioidosis was defined by culture of B. pseudomallei in any clinical sample. Other infection cases were defined as detection of other pathogenic microorganisms by culture and laboratory testing, including molecular tests, microscopy, lateral flow chromatographic immunoassay, or electrochemiluminescence immunoassay at the Mukdahan Hospital clinical laboratory. A no-pathogen-detected group was defined as no pathogenic microorganism identified by all laboratory tests.

### Clinical samples.

Clinical samples, including blood, pus, sputum, urine, and peritoneal dialysis fluid (PD fluid), were collected and processed at the study site. Five milliliters of blood was obtained in an EDTA tube. Whole blood was centrifuged at 1,500 × *g* for 15 min, and plasma and buffy coat fractions were collected. The buffy coat was lysed with an equal volume of sterile ultrapurified water. Pus was obtained using a disposable sterile cell harvester (Jiangsu Jianyou Medical Technology, JiangSu, China) and resuspended in 500 μL of sterile phosphate-buffered saline. Sputum was collected and mixed with an equal volume of sterile 4% NaOH.

Ten milliliters of blood was cultured using BacT/Alert 3D (bioMérieux, Marcy l'Étoile, France), and urine was cultured on Ashdown agar (23), sheep blood agar, and MacConkey agar. Suspected B. pseudomallei colonies were tested with a B. pseudomallei-specific latex agglutination test (24). Biochemical identification and antibiotic susceptibility testing were performed as described in ASM’s *Clinical Microbiology Procedures Handbook* (25). All clinical samples, including urine, plasma, buffy coat, pus, sputum, and peritoneal dialysis fluid, were stored at −80°C until used for TTS1 real-time PCR, Hcp1-ELISA, and OPS-ELISA.

### Hcp1-ICT.

**FIG 2:**
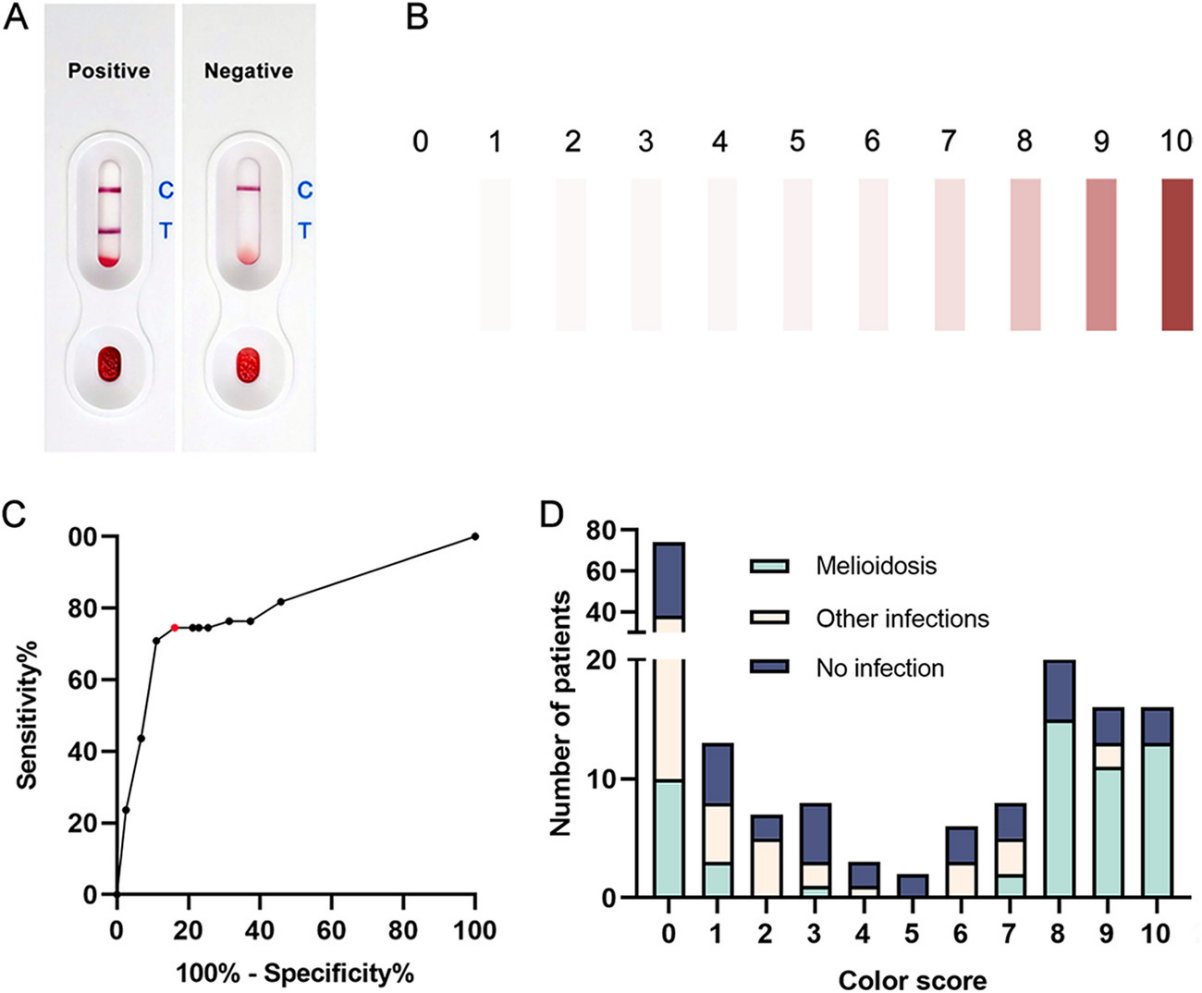
Hcp1-ICT tests. (A) Hcp1-ICT results with whole blood samples. (B) Color score of Hcp1-ICT results. (C) The receiver operating characteristic (ROC) curve was estimated using proportions of bacterial cultures of patient showing scores of 0 to 10 in the Hcp1-ICT. Hcp1-ICT tests with the color score at test bands 7 to 10 were considered positive. The absence of test bands and a color score of 1 to 6 were interpreted as negative. The red dot represents the cutoff score at 7. (D) Number of patients with melioidosis, other infections, and no pathogenic infection for each color score of Hcp1-ICT results.

The Hcp1-ICT (lot number 19F1003) was performed with whole blood samples as previously described (17) (Fig. 2A). In brief, a 10-μL EDTA-blood sample was applied to the sample well, followed by 4 drops of running buffer. The result was read following 10 min of incubation at room temperature and interpreted by the presence of a control line. Prior to knowledge of culture results, the intensity of the test line color was assigned a score from 0 to 10 (Fig. 2B). Subsequently, receiver operating characteristic (ROC) curve analysis was performed to optimize the area under the curve. Based on this analysis, scores of 7 to 10 were interpreted as positive results. In contrast, the absence of any test line (score of 0) and scores of 1 to 6 were interpreted as negative. All scores were confirmed by 3 independent examiners.

### DNA extraction.

DNA was extracted from 2 mL of plasma sample using a QIAamp DNA blood midi kit (Qiagen, Hilden, Germany) as recommended by the manufacturer with a final elution volume of 140 μL. Buffy coat obtained from 5 mL of EDTA-blood or 200 μL of other clinical samples, including uncentrifuged urine, peritoneal dialysis fluid, pus, and sputum, was DNA extracted using a QIAamp DNA Mini Kit per the manufacturer’s instructions (Qiagen, Hilden, Germany) with a final elution volume of 70 μL.

The control for DNA extraction was *orf2* of the B. pseudomallei TTS1 (8). The 115-bp PCR product was used as a template with the sequence modification at the probe hybridization location. This region was substituted with double-strand oligonucleotides of Angiostrongylus vasorum cytochrome *c* oxidase subunit I. The fragment was synthesized and ligated into pUC57 plasmid as TTS1-AVa (Bionic, South Korea). Plasmids were transformed into Escherichia coli JM110 for propagation. TTS1-AVa DNA was obtained using the QIAprep Spin Miniprep kit (Qiagen, Hilden, Germany). In each sample, 2.5 pg of TTS1-AVa DNA was added as an internal control prior to DNA extraction.

### Real-time PCR.

The real-time PCR assay targeted the 115-bp *orf2* of B. pseudomallei TTS1 (8). Each reaction mixture consisted of a total volume of 11 μL with 1× SensiFAST probe No-ROX (Bioline, Australia), 500 nM (each) BpTT4176F primer (5′-GCTCTCTATACTGTCGAGCAATGC-3′) and BpTT4290R primer (5′-CGTGCACACCGGTCAGTATC-3′), 200 nM BpTT4208P probe (5′–6-carboxyfluorescein [FAM]–CCGGAATCTGGATCACCACCACTTTCC–black hole quencher 1 [BHQ1]–3′) (8), 200 nM TTS1-AVa probe (5′-hexachlorofluorescein [HEX]-CTCCGTTGAGTAGTTTGGGTCATCCGG-BHQ1 3′), and 4 μL of total genomic DNA template. The thermal cycling conditions were 95°C for 5 min, followed by 95°C of 15 s and 59°C of 30 s for 45 cycles. The positive control was 100 pg of B. pseudomallei K96243 DNA. Real-time PCR was performed on a CFX96 Touch real-time PCR system (Bio-Rad, Hercules, CA).

Results of the Hcp1-ICT and TTS1 real-time PCR were analyzed as a combined test for antibody and DNA detection. A positive combined result was defined when either the Hcp1-ICT or TTS1 real-time PCR was positive, and a negative combined result was reported when both Hcp1-ICT and TTS1 real-time PCR results were negative.

### Hcp1-ELISA and OPS-ELISA.

ELISAs were performed on plasma samples at a dilution of 1:250, using recombinant Hcp1 (15, 26) at 2.5 μg/mL and OPS (27) at 1 μg/mL, with a 1:2,000 dilution of horseradish peroxidase-conjugated rabbit anti-human IgG as the secondary antibody (14). All samples were performed in duplicate. The results were determined using a Sunrise microplate reader (Tecan, Männedorf, Switzerland) at an optical density of 450 nm (OD_450_). The samples were considered positive if the OD was ≥1.165 for Hcp1-ELISA and ≥0.875 for OPS-ELISA, as previously described (15).

### Statistical analyses.

Statistical analyses were performed using Stata version 14 (Stata Corp. LP, College Station, TX, USA) and Prism 8 statistics (GraphPad Software Inc., La Jolla, CA). The continuous variables and proportions for discrete data were presented as the median and interquartile range (IQR). IQRs were presented for 25th and 75th percentiles. Data were compared between groups using the Kruskal-Wallis test and the chi-square test for categorical data, continuous variables, and proportions. The data in the box plot demonstrate the 25th to 75th percentiles, with the middle line representing the median. The whiskers indicate the 10th and 90th percentiles. The sensitivity, specificity, positive predictive value (PPV), and negative predictive value (NPV) of all tests were calculated using bacterial culture results as a gold standard. The McNemar test was used to compare the sensitivity and specificity between tests.

## RESULTS

### Characteristics of participants.

A total of 180 adult patients with suspected acute melioidosis were screened in the study (Fig. 1). Seven individuals were excluded because they were repeat cases (*N* = 6) or referred from other hospitals after ≥72 h (*N* = 1). The final number of patients enrolled and analyzed was 173. Of these patients, initial diagnostic testing using bacterial culture identified 54 (31.2%) confirmed melioidosis patients. Forty six (26.6%) patients were diagnosed with infection due to other pathogens, and 73 (42.2%) patients had no identified infection by bacterial culture or by testing for infections using other methods. The 73 patients without identified infection were subjected to further testing as described in Fig. 1. Extended incubation of blood culture samples to 15 days did not result in identification of B. pseudomallei; however, one additional patient had a bacterial culture that was positive for B. pseudomallei from the clinical sample collected on day 4. Day 4 sampling also identified three other infectious etiologies. In total, 55 (31.8%) patients were classified as having melioidosis, 49 (28.3%) patients were classified as having other infections, and 69 (39.9%) patients had no diagnosed etiology of infection.

Table 1 shows the demographic and clinical characteristics of the patients. The median age was 58 years (IQR, 47 to 68 years) for all patients, 55 years (IQR, 45 to 62 years) for the melioidosis group, 64 years (52 to 71 years) for the other infections group, and 57 years (IQR, 48 to 67 years) for the no-infection group (*P* = 0.03). Seventeen melioidosis cases (30.9%) were admitted to the intensive care unit (ICU). Patients in the melioidosis group had longer hospitalizations than patients from other groups. The median time of hospitalization in the melioidosis group was 14 days (IQR, 11 to 20 days), compared with 12 days for the other infections group (IQR, 5 to 14 days) and 5 days for the no-pathogen-detected group (IQR, 3 to 8.5 days) (*P* < 0.001).

**TABLE 1:** Characteristics of patients with suspected melioidosis in this study

Characteristic	Total (*N* = 173)	Melioidosis (*N* = 55)	Other infections (*N* = 49)	No pathogen detected (*N* = 69)	*P* value^*a*^
Median age (IQR)	58 yrs (47–68)	55 yrs (45–62)	64 yrs (52–71)	57 yrs (48–67)	0.03
Gender					0.37^*b*^
Male (%)	92 (53.2)	32 (58.2)	22 (44.9)	38 (55.1)	
Female (%)	81 (46.8)	23 (41.8)	27 (55.1)	31 (44.9)	
28-day mortality	14 (8.1%)	5 (9.1%)	3 (5.8%)	6 (9.1%)	0.35
No. (%) with previous history of melioidosis	10 (5.8%)	6 (10.9%)	1 (1.9%)	3 (4.5%)	0.15
No. (%) admitted to ICU	36 (20.8%)	17 (30.9%)	9 (18.4%)	10 (14.5%)	0.22
Median no. of days hospitalized (IQR)	9 (4.0–14.0)	14 (11–20)	12 (5–14)	5 (3.0–8.5)	<0.001

a Statistical testing of the demographics among three groups was performed with the Kruskal-Wallis test.

b Statistcal testing of the demographics among the three groups was performed with a chi-square test.

### Optimization of the Hcp1-ICT test cutoff.

All Hcp1-ICT results performed on whole blood samples on admission were determined by assigning a score based on the color of the test band for patients before culture results were known (Fig. 2B). Combining this information and the subsequent identification of melioidosis patients using bacterial culture, ROC analysis was performed to identify the optimal cutoff for Hcp1-ICT interpretation on whole blood samples. Assigning a score of ≥7 as positive yielded a test sensitivity of 74.6% and specificity of 83.9%, with AUC of 0.80 (Fig. 2C). Based on this, we decided to interpret Hcp1-ICT as positive at a color score of ≥7. A majority of melioidosis patients (41; 74.6%) were noted to be Hcp1-ICT positive, with scores of 7 to 10. In contrast, the absence of test bands was noted for most patients with other infections (28; 57.1%) or patients with no infection (36; 52.2%). However, some patients with other infections or no infection were scored as false positive by Hcp1-ICT at a color score of ≥7 (Fig. 2D).

### Diagnostic accuracy of Hcp1-ICT using whole blood samples.

Using the threshold of color score at test band 7 to dichotomize the Hcp1-ICT results as positive or negative, other measures of diagnostic accuracy (sensitivity, specificity, PPV, and NPV) were assessed (Table 2). The Hcp1-ICT was positive in 60 of 173 (34.7%) patients with suspected melioidosis in the study. The sensitivity of Hcp1-ICT for 55 culture-confirmed melioidosis patients was 74.5% (95% confidence interval [CI], 61.7% to 84.2%). The specificity for 49 patients with other infections was 89.8% (95% CI, 78.2% to 95.6%) and for 69 patients with no pathogenic infection it was 79.7% (95% CI, 68.8% to 87.5%). Using all 173 patients, the PPV and NPV of Hcp1-ICT were 68.3% (95% CI, 55.8% to 78.7%) and 87.6% (95% CI, 80.3% to 92.5%), respectively.

**TABLE 2:** Diagnostic accuracy of the tests used in this study

Test	% Sensitivity (95% CI)	% Specificity for other infections group (95% CI)	% Specificity for no-pathogen-detected group (95% CI)	% PPV (95% CI)	% NPV (95% CI)
Hcp1-ICT	74.5 (61.7–84.2)	89.8 (78.2–95.6)	79.7 (68.8–87.5)	68.3 (55.8–78.7)	87.6 (80.3–92.5)
TTS1 real-time PCR	78.2 (65.6–87.1)	100.0 (92.7–100.0)	98.6 (92.2–99.9)	97.7 (88.2–99.9)	90.7 (84.4–94.6)
Combined Hcp1-ICT and TTS1 real-time PCR	98.2 (90.4–99.9)	89.8 (78.2–95.6)	78.3 (67.2–86.4)	73.0 (61.9–81.8)	99.0 (94.5–99.9)
Hcp1-ELISA	70.9 (57.9–81.2)	85.7 (73.3–92.9)	76.8 (65.6–85.2)	62.9 (50.5–73.8)	85.6 (77.9–90.9)
OPS-ELISA	69.1 (56.0–79.7)	89.8 (78.2–95.6)	68.1 (56.4–77.9)	58.5 (46.3–69.6)	84.3 (76.2–89.9)

The Hcp1-ICT was positive in 5 of 49 (10.2%) of the patients with other infections (Table 3), as follows: Escherichia coli (2/17 patients), Klebsiella pneumoniae (2/4 patients), and Pseudomonas aeruginosa (1/1 patient). The patient infected with P. aeruginosa had a history of melioidosis 37 weeks prior to enrollment in this study.

**TABLE 3:** Infectious agents isolated from clinical samples of 49 with other infections

Infectious agent	Total no. of patients	Hcp1-ICT positive	Hcp1-ELISA positive	OPS-ELISA positive	TTS1 real-time PCR positive
Acinetobacter baumannii	3	0	0	0	0
Aeromonas hydrophila	1	0	0	0	0
Brucella spp.	1	0	0	0	0
*Corynebacterium* spp.	1	0	0	0	0
Enterobacter cloacae	2	0	0	0	0
Enterococcus faecalis	5	0	0	0	0
Enterococcus faecium	1	0	0	0	0
Escherichia coli	17	2	3	2	0
Klebsiella pneumoniae	4	2	2	1	0
Pseudomonas aeruginosa	1	1	1	1	0
Staphylococcus aureus	2	0	0	0	0
Streptococcus pyogenes	2	0	0	0	0
Mixed infection	9	0	1	1	0
Total	49	5	7	5	0

### Sensitivity of diagnostic tests in culture-confirmed melioidosis patients.

**FIG 3:**
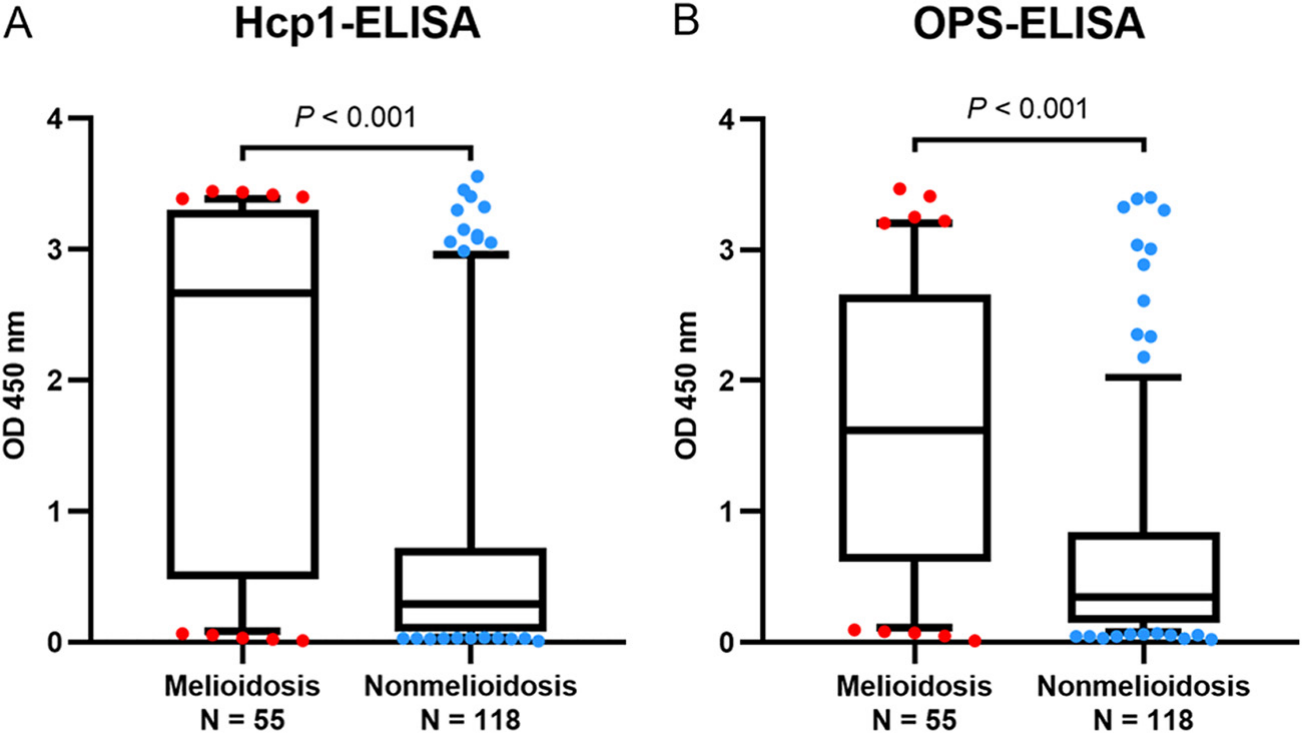
ELISA results for melioidosis and nonmelioidosis patients. (A) Hcp1-ELISA. (B) OPS-ELISA. The box plots represent OD450 readings and extend from the 25th to 75th percentiles, with the middle lines representing the medians. The whiskers indicate the 10th and 90th percentiles.

The performance of the Hcp1-ICT for diagnosis of melioidosis for 173 suspected cases was compared with the results of TTS1 real-time PCR, Hcp1-ELISA, and OPS-ELISA (Table 2). The real-time PCR was performed on DNA extracted from various clinical samples (*N* = 543) of 173 patients. Hcp1-ELISA and OPS-ELISA were performed on plasma samples from all 173 patients. All samples were collected from patients on the first day of admission. The sensitivities of TTS1 real-time PCR, Hcp1-ELISA, and OPS-ELISA in 55 culture-confirmed melioidosis patients were 78.2% (95% CI, 65.6 to 87.1%), 70.9% (95% CI, 57.9 to 81.2%), and 69.1% (95% CI, 56.0 to 79.7%), respectively. The median OD values of the Hcp1-ELISA and the OPS-ELISA in culture-confirmed melioidosis patients were higher than for nonmelioidosis patients (Hcp1-ELISA: 2.669 with IQR of 0.482 to 3.302, compared to results in nonmelioidosis patients, 0.290 with IQR of 0.078 to 0.718; *P* < 0.001; OPS-ELISA: 1.622 with IQR of 0.620 to 3.468 versus 0.341 with IQR o 0.144 to 0.843; *P* < 0.001) (Fig. 3A and B).

The sensitivity of the Hcp1-ICT was not significantly different from that for the TTS1 real-time PCR (74.5% versus 78.2%; *P* = 0.839), the Hcp1-ELISA (74.5% versus 70.9%; *P* = 0.500), or the OPS-ELISA (74.5% versus 69.1%; *P* = 0.453). The combined results of the Hcp1-ICT and TTS1 real-time PCRs were analyzed, and the sensitivity increased to 98.2% (95% CI, 90.4 to 99.9%), which was higher than that of the Hcp1-ICT alone (74.5%; *P* < 0.001), TTS1 real-time PCR alone (78.2%; *P* < 0.001), Hcp1-ELISA (70.9%; *P* < 0.001), or OPS-ELISA (69.1%; *P* < 0.001) (Table 2). TTS1 real-time PCR was positive in 13 of 15 (86.7%) culture-confirmed melioidosis patients who were Hcp1-ICT negative.

### Specificity of the diagnostic tests used in this study in nonmelioidosis patients.

The specificities of the diagnostic tests used in this study were first calculated for results with the 49 patients with other infections. The specificity of TTS1 real-time PCR was the highest, at 100% (95% CI, 92.7 to 100%) (Table 2). The specificity of the Hcp1-ICT was 89.8% (95% CI, 78.2 to 95.6%), which was comparable to that for the TTS1 real-time PCR (*P* = 0.063), the Hcp1-ELISA (85.7%, with 95% CI of 73.3 to 92.9%; *P* = 0.500), and the OPS-ELISA (89.8%, with 95% CI of 95.6%; *P* = 1.000). The specificity of the combined Hcp1-ICT and TTS1 real-time PCR tests was 89.8% (95% CI, 78.2 to 95.6%), which was comparable to that for Hcp1-ICT alone (89.8%; *P* = 1.000), the Hcp1-ELISA (85.7%; *P* = 0.500), or the OPS-ELISA (89.8%; *P* = 1.000), but lower than that for the TTS1 real-time PCR alone (100%; *P* = 0.063).

The specificity in the no-pathogen-detected group (69 patients) was highest for TTS1 real-time PCR at 98.6% (95% CI, 92.2 to 99.9%). The Hcp1-ICT specificity for this group was 79.7% (95% CI, 68.8 to 87.5%), compared to that for the TTS1 real-time PCR (*P* = 0.001), the Hcp1-ELISA (76.8%, with 95% of CI 65.6 to 85.2%; *P* = 0.625) or the OPS-ELISA (68.1%, with 95% CI of 56.4 to 77.9%; *P* = 0.057). The combined test (Hcp1-ICT + TTS1 real-time PCR) specificity was 78.3% (95% CI, 67.2 to 86.4%), compared to 79.7% for the Hcp1-ICT (*P* = 1.000), 76.8% for the Hcp1-ELISA (*P* = 1.000), 68.1% for the OPS-ELISA (*P* = 0.119), and 98.6% for TTS1 real-time PCR (*P* < 0.001).

### Sensitivity of the diagnostic tests used in this study for melioidosis patients with bacteremia.

Of 55 melioidosis patients, 38 (69.1%) patients were positive for B. pseudomallei from blood culture. TTS1 real-time PCR sensitivity was the highest (78.9%, 95% CI, 63.7 to 88.9%) among the four single tests used (Table 4). The sensitivity of the Hcp1-ICT, Hcp1-ELISA, and OPS-ELISA were 73.7% (95% CI, 58.0 to 85.0%), 71.1% (95% CI, 55.2 to 83.0%), and 63.2% (95% CI, 47.3 to 76.6%), respectively.

**TABLE 4:** Sensitivity of the diagnostic tests used in this study in melioidosis patients with bacteremia or abscesses

Infection type	Sensitivity (%) of test (95% CI)				
	Hcp1-ICT	TTS1 real-time PCR	Combined Hcp1-ICT and TTS1 real-time PCR	Hcp1-ELISA	OPS-ELISA
Bacteremia	73.7 (58.0–85.0)	78.9 (63.7–88.9)	97.4 (86.5–99.9)	71.1 (55.2–83.0)	63.2 (47.3–76.6)
Abscess	84.6 (57.8–97.3)	69.2 (42.4–87.3)	100 (77.2–100.0)	76.9 (49.7–91.8)	84.6 (57.8–97.3)

The combined results of the Hcp1-ICT and TTS1 real-time PCR tests demonstrated a sensitivity of 97.4% (95% CI, 86.5 to 99.9%) in bacteremic melioidosis patients. The sensitivity of the combined tests was greater than that for the Hcp1-ICT alone (73.7%; *P* = 0.004), TTS1 real-time PCR alone (78.9%; *P* = 0.016), Hcp1-ELISA (71.1%; *P* = 0.002), and the OPS-ELISA (63.2%; *P* < 0.001).

### Positivity rate of the diagnostic tests used in this study for melioidosis in patients with abscesses.

Of 21 patients with abscesses in their skin or internal organs, 13 were positive for melioidosis (61.9%), 1 belonged to the other infections group (4.8%), and 7 belonged to the no-pathogenic-infection group (33.3%). Ten of 21 (47.6%) patients had imaging for internal abscesses with a computed tomography (CT) scan of the abdomen or abdominal ultrasound. The abscesses in the 13 melioidosis patients included 9 skin abscesses (69.2%), 3 splenic abscesses (23.1%), and 1 hepatosplenic abscess (7.7%). One patient from other infections group had a skin abscess. In 7 patients with no pathogenic infection, we detected 1 hepatosplenic abscess (14.3%), 4 hepatic abscesses (57.1%), 1 lung abscess (14.3%), and 1 lymph node abscess (14.3%).

The combined Hcp1-ICT and TTS1 real-time PCR test results presented the highest positivity rate, at 13/13 (100%) in any clinical sample from melioidosis patients with abscesses, including urine, plasma, buffy coat, sputum, and pus, followed by the Hcp1-ICT (11/13; 84.6%) and the OPS-ELISA (11/13; 84.6%), the Hcp1-ELISA (10/13; 76.9%), and TTS1 real-time PCR (9/13; 69.2%) (Table 4). The clinical specimens that were TTS1 real-time PCR positive for these patients included 6/13 urine samples (46.2%), 1/13 plasma samples (7.7%), 3/13 buffy coat samples (23.1%), 3/3 sputum samples (100%), and 4/4 pus samples (100%).

### Positivity of the diagnostic tests used in this study and number of days post-symptom onset in bacteremic melioidosis patients.

**FIG 4:**
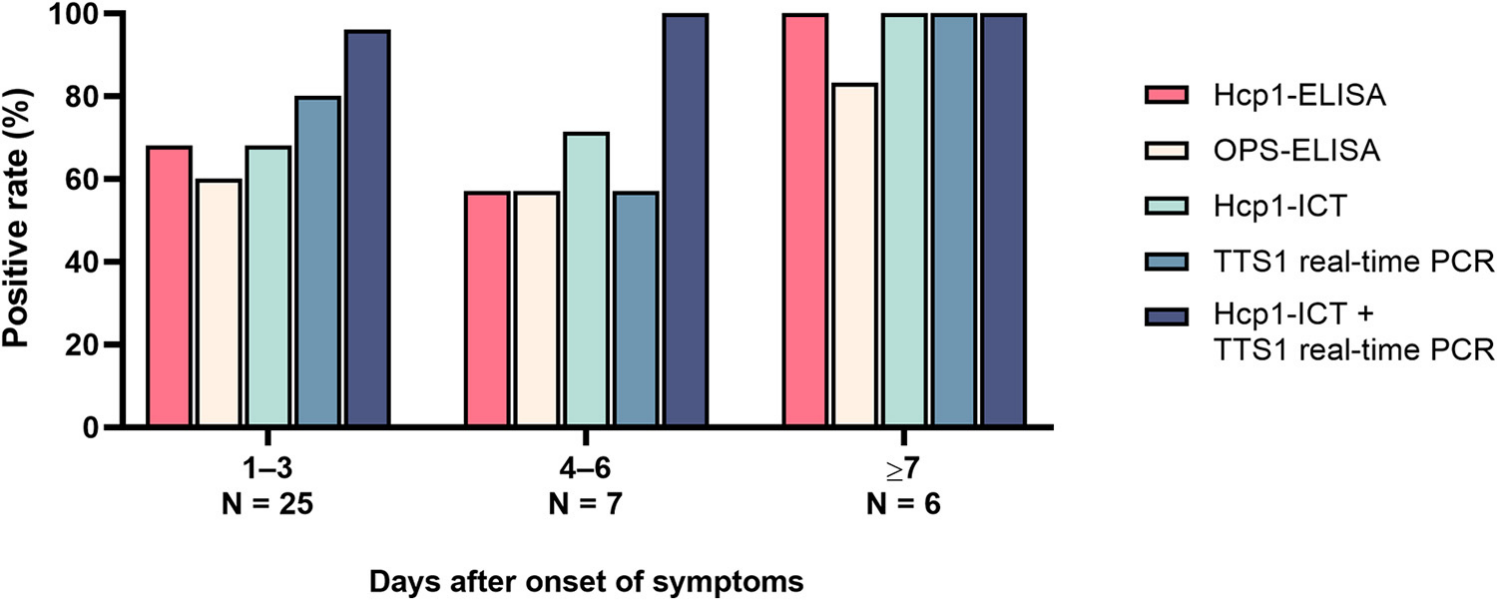
The positivity rate of the diagnostic tests used in this study at different periods post-symptom onset among bacteremic melioidosis patients.

Thirty-eight patients were documented with bacteremic melioidosis. Of these, 25 (65.8%) patients had symptoms for 1 to 3 days, 7 patients (18.4%) had symptoms for 4 to 6 days, and 6 patients (15.8%) had symptoms for ≥7 days. The combination of Hcp1-ICT and TTS1 real-time PCR tests presented the highest positivity rates, 96% at 1 to 3 days and 100% at more than 4 days, compared with any single test. At 1 to 3 days post-symptom onset, every single test presented a positivity rate ranging from 60% to 80%. At 4 to 6 days post-symptom onset, the detection rate for single tests was 57.1% for the Hcp1-ELISA, the OPS-ELISA, and TTS1 real-time PCR and 71.4% for the Hcp1-ICT. The positivity rates of all tests were highest at ≥7 days, with 100% for the Hcp1-ICT, TTS1 real-time PCR, and Hcp1-ELISA, followed by 85.7% for the OPS-ELISA (Fig. 4).

### TTS1 real-time PCR detection in melioidosis patients.

**FIG 5:**
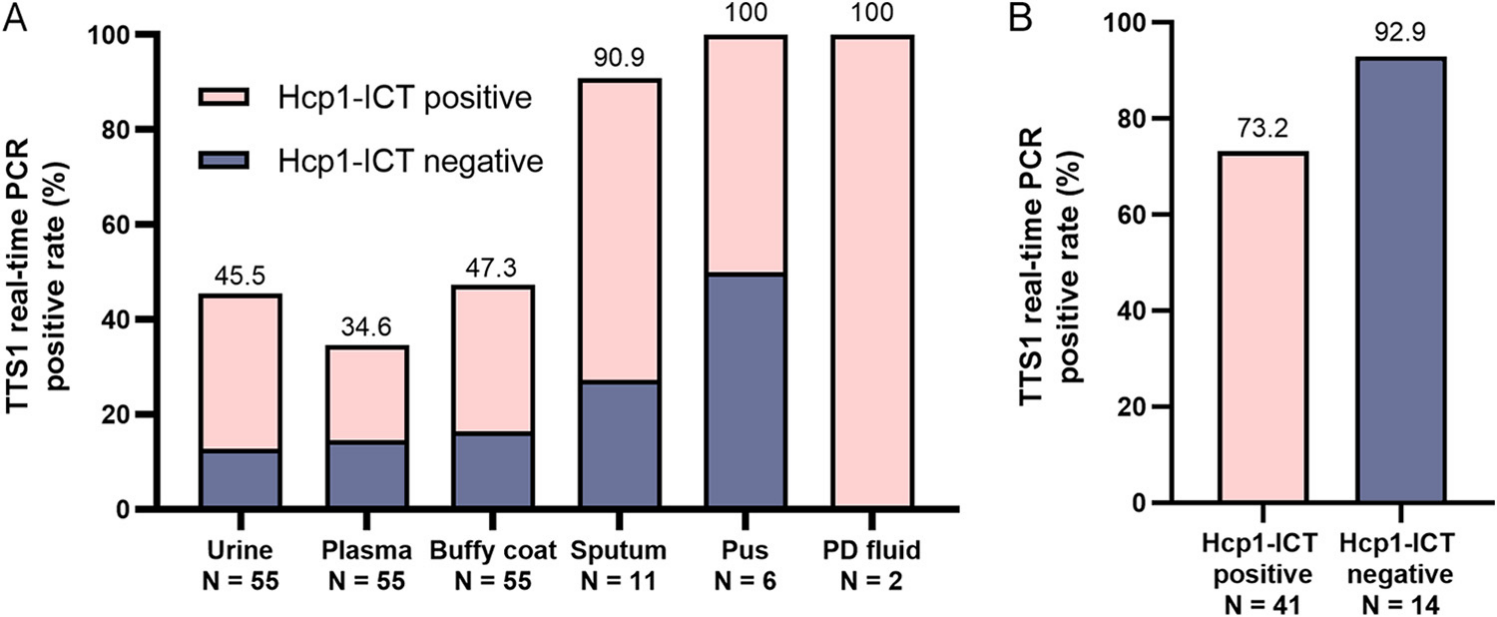
Positivity rate of TTS1 real-time PCR in melioidosis patients. (A) The positivity rate of TTS1 real-time PCR in different types of clinical samples. (B) Positivity rate of TTS1 real-time PCR associated with Hcp1-ICT results in melioidosis patients.

A total of 543 clinical samples were collected from 173 patients on the day of admission with a range of 3 to 4 samples each (median, 3 samples per patient). Of 55 melioidosis patients, 184 clinical samples were collected. The positivity rate of TTS1 real-time PCR in melioidosis patients was 34.6% (19/55) in plasma samples, 47.3% (26/55) in buffy coat samples, 45.5% (25/55) in urine samples, 90.9% (10/11) in sputum samples, 100% (6/6) in pus samples, and 100% (2/2) PD fluid samples. The melioidosis patients that were TTS1 real-time PCR positive included patients who were both Hcp1-ICT positive and negative (Fig. 5A).

Of 41 Hcp1-ICT-positive melioidosis patients, TTS1 real-time PCR was positive in 73.2% (30/41) of any clinical samples (Fig. 5B). The positivity rate was 43.9% (18/41) in urine samples, 26.8% (11/41) in plasma samples, 41.5% (17/41) in buffy coat samples, 87.5% (7/8) in sputum samples, 100% (3/3) in pus samples, and 100% (2/2) in PD fluid samples.

Of 14 Hcp1-ICT-negative melioidosis patients, we found that TTS1 real-time PCR was 92.9% (13/14) positive in any clinical sample (Fig. 5B). The positivity rate for each sample type was 50% (7/14) in urine samples, 57.1% (8/14) in plasma samples, 64.3% (9/14) in buffy coat samples, 100% (3/3) in sputum samples, and 100% (3/3) in pus samples.

### Hcp1-ICT for identification of occult melioidosis patients.

Culture results of the first samples from 73 patients collected at admission were negative. Sixteen of these patients were Hcp1-ICT positive. Eight of 16 (50%) culture-negative Hcp1-ICT-positive patients were either subsequently culture positive for B. pseudomallei infection (*N* = 5; 31.3%) or had clinical features that were especially suggestive of melioidosis (*N* = 3; 18.8%) (Table 5). Of the five patients with delayed confirmation of infection, one was culture-positive from a blood sample collected on day 4 of admission as per the study protocol. Four reported no significant illness recovery after discharge from the hospital and were readmitted to the same hospital for culture-confirmed B. pseudomallei infection 3 to 11 weeks after the first admission, which suggests that melioidosis caused the initial illness. Of the patients with suggestive clinical features, one patient with diabetes mellitus, alcoholism, and prolonged fever was diagnosed with probable melioidosis by the treating clinicians, based on diagnostic imaging, including CT of the upper abdomen and ultrasound of the whole abdomen, which showed liver and spleen abscesses with pleural effusion. Additionally, two patients were deemed to be possible occult cases of melioidosis, as they had previously been diagnosed with melioidosis 3 months and 15 months prior to enrollment in this study.

**TABLE 5:** Potential of the Hcp1-ICT for detection of occult cases of melioidosis among patients with initially negative cultures

Hcp1-ICT result	No. (%) of occult melioidosis patients with indicated Hcp1-ICT result, by finding on follow-up			
	Confirmed melioidosis	Probable or possible melioidosis		
	B. pseudomallei positive in repeated culture	Clinical finding^*a*^	Previous melioidosis	TTS1 real-time PCR
Positive (*N* = 16)	5 (31.3%)	1 (6.3%)	2 (12.5%)	0
Negative (*N* = 57)	0	0	0	1 (1.8%)
Total (*N* = 73)	5 (6.9%)	1 (1.4%)	2 (2.7%)	1 (1.4%)

a Clinical finding was diagnosed by the treating clinician based on the clinical picture, CT scan, and abdominal ultrasound results.

Fifty-seven of the 69 patients (78.1%) in the no-pathogen-detected group were Hcp1-ICT negative. No patients converted to Hcp1-ICT positive on repeat assessment on day 4.

Of the 16 Hcp1-ICT-positive patients without evidence of infection upon enrollment sampling, 15 (93.8%) had positive Hcp1-ELISA and 13 (81.3%) had positive OPS-ELISA. None of these patients were positive by TTS1 real-time PCR performed on the buffy coat, plasma, or urine samples. Nine patients (56.3%) underwent repeat Hcp1-ICT on day 4 and all were still positive.

## DISCUSSION

In melioidosis, early diagnosis is critical to initiate prompt treatment with effective antibiotics, prevent disease progression, and improve outcomes. While bacterial culture is the standard method for diagnosis, it is time-consuming and provides low sensitivity (3). In this study, we evaluated the Hcp1-ICT as a point-of-care test, and we prospectively evaluated the diagnostic accuracy of antibody tests, including the Hcp1-ICT, the Hcp1-ELISA, the OPS-ELISA and TTS1 real-time PCR tests, performed within 24 h of admission of suspected melioidosis patients in northeast Thailand. This study demonstrated that a combination of the Hcp1-ICT and TTS1 real-time PCR tests could be useful tools for rapidly identifying B. pseudomallei with high sensitivity and specificity. The diagnostic accuracy of the combined tests was higher than that for any single test, and we determined that the Hcp1-ICT alone may help increase clinical suspicion for melioidosis in culture-negative individuals.

Reading the color intensity of the Hcp1-ICT test bands as scores ranging from 1 to 10 has provided an interpretation of IgG antibody levels in patients. Our evaluation using whole blood samples indicated a high potential when using scores of ≥7 as positive. However, the interpretation of the visible test bands could lead to an interpretation error, especially when difficult-to-read bands are nearly at the cutoff score. Our future studies will evaluate a mobile application to distinguish between positive and negative results. Artificial intelligence was recently applied to improve the interpretation of lateral flow assay for 2019 coronavirus disease detection (28). Using whole blood samples is ideal for POC tests at the bedside, because this does not require centrifugation to separate serum or plasma. The sensitivity of the Hcp1-ICT evaluated with whole blood samples was 74.5%, lower than that previously reported using serum samples (88.3%) (17). The lower sensitivity was possibly due to differences in the designs of the two studies. In this study, we prospectively collected blood samples on the day of admission. The previous study collected samples at least 48 to 72 h after melioidosis patient identification by culture results. Our data showed improved sensitivity when the result of the Hcp1-ICT was combined with the result of TTS1 real-time PCR. The data in this study support the idea that TTS1 real-time PCR could identify melioidosis patients with slow or no IgG seroconversion when their specimens are collected early in infection and the Hcp1-ICT was negative.

The sensitivity of the TTS1 real-time PCR was high for sputum, pus, urine, and PD fluid samples, with high bacterial loads as previously reported (10, 11). As expected, blood samples, including buffy coat and plasma, showed less sensitivity by TTS1 real-time PCR (10, 11). A quantitative blood culture study showed that the median concentration of B. pseudomallei in blood samples was 1.1 CFU/mL (29). The sensitivity of TTS1 real-time PCR in 200-μL blood samples evaluated in Thailand was 0% (10). In this study, we used a high-volume blood samples (5 mL) and eluted the DNA with a low buffer volume (70 μL for buffy coat and 140 μL for plasma), which improved sensitivity in the buffy coat to 47.3% and 34.6% in plasma. However, the sensitivity of TTS1 real-time PCR in human whole blood samples may be low due to the presence of PCR inhibitors, such as immunoglobulin G, heme, and human leukocyte DNA (6, 7). Our study showed that the sensitivity of TTS1 real-time PCR using 2 mL of plasma was not as high as using buffy coat samples. Using a higher volume of plasma with a small volume of elution buffer might improve the sensitivity of testing blood samples. Uncentrifuged urine was used in this study because a previous study showed no difference in results between using 10 mL of centrifuged urine and 200 μL of uncentrifuged urine (30). TTS1 real-time PCR detection with the samples collected from localized infections with B. pseudomallei, such as pus samples, might be useful, as the sensitivity of TTS1 real-time PCR in melioidosis patients was 100%. Unfortunately, we were not able to collect pus from all melioidosis patients with abscesses, because pus samples were not available for some patients, including those with internal organ infections. The TTS1 real-time PCR assay could lead to a false-negative result by using a single target for detection. Combining the TTS1-*orf2* with other targets, such as BPSS0745, could potentially improve the sensitivity of these assays, as demonstrated previously in the detection of B. pseudomallei DNA in soil samples, where the sensitivity increased from 76.5% to 90% (9).

TTS1 real-time PCR-positive results in patients with symptom onset of ≥7 days might be related to several potential factors, including persistent infection. B. pseudomallei persistence has been reported to be associated with toxin-antitoxin systems, the ability of bacteria to survive under stressful conditions, and adaptive mutations (31). Furthermore, the response of B. pseudomallei to initial intensive therapy could be slow, as median fever clearance time is 9 days (32). A longer response time is observed in patients with deep-seated abscesses (1). PCR detection could also be positive after the fever clearance phase due to DNA from dead cells.

The sensitivity and specificity of serological testing, including the Hcp1-ICT, the Hcp1-ELISA, and the OPS-ELISA were less than those reported in the previous study (15). Lower accuracy could have been due to different times of sample collection. This study collected samples within the first 24 h of patient admission. Another explanation is that the two studies were conducted in different study populations. Our study enrolled one population of suspected melioidosis patients from Mukdahan Hospital, while various populations, including Thai healthy donors, U.S. healthy donors, tuberculosis patients, scrub typhus patients, and leptospirosis patients were included in the previous study (15). We also determined that 5.1% of nonmelioidosis patients had previous infections with B. pseudomallei. Serological tests detected IgG antibodies against Hcp1 and OPS in 83.3% and 100% of samples tested, respectively, from nonmelioidosis patients with previous infections. The patients were infected 60 days to 20 months prior to this study. The IgG antibodies against Hcp1 and OPS in melioidosis patients were found 3 to 155 days of duration of symptoms before admission (16). The detectable level of IgG antibodies benefits early detection of Hcp1-ICT in melioidosis patients with <7 days post-symptom onset, as we found the positivity rate was 68.8%.

Blood cultures were positive in approximately 50% of melioidosis patients (32), and time to result for B. pseudomallei report in culture could be up to 4 days (1). However, among patients with initially negative cultures, 31% of patients with a positive Hcp1-ICT subsequently had culture-confirmed B. pseudomallei infections. Since the Hcp1-ICT has a turnaround time of 15 min, it may be a useful tool in prompting clinicians to consider melioidosis as a diagnosis among patients with unidentified infections (17).

The evaluation of the different diagnostic tests in this study showed that the Hcp1-ICT is a promising test for detection of melioidosis, since it demonstrated high sensitivity, high specificity, and a short turnaround time. Using the Hcp1-ICT is rapid and simple. It does not require specific training or equipment. However, the limitations of the Hcp1-ICT are that it cannot distinguish between IgG antibody responses to previous and current infections. Hcp1-ICT can be improved by combining with TTS1 real-time PCR for antigen and antibody detection. Based on our findings, the combination of the Hcp1-ICT and TTS1 real-time PCR could be a rapid diagnostic test for early diagnosis in clinical settings and for surveillance of melioidosis and may also facilitate the identification of initially occult melioidosis.
